# Nature-inspired recycling of a protein mixture into a green fluorescent protein-based hydrogel†

**DOI:** 10.1039/d4su00212a

**Published:** 2024-07-23

**Authors:** Laura Roset Julià, Sebastian J. Maerkl, Francesco Stellacci

**Affiliations:** a Institute of Materials, Ecole Polytechnique Fédérale de Lausanne (EPFL) 1015 Lausanne Switzerland francesco.stellacci@epfl.ch; b NCCR Bio-Inspired Materials, Ecole Polytechnique Fédérale de Lausanne (EPFL) 1015 Lausanne Switzerland; c Bioengineering Institute, Ecole Polytechnique Fédérale de Lausanne (EPFL) 1015 Lausanne Switzerland; d Global Health Institute, Ecole Polytechnique Fédérale de Lausanne (EPFL) 1015 Lausanne Switzerland

## Abstract

Protein-based materials are biocompatible and have a variety of remarkable properties; consequently, they are finding more and more applications. Nature recycles proteins in multiple ways, ranging from bio-degradation (a slow approach) to fast recycling of protein metabolism. The latter is a wonderful example because a random mixture of proteins gets digested into amino acids (AAs), the fundamental building blocks of proteins. These AAs are then used by cells to produce whichever protein is needed at the time of synthesis. Seen through the lens of recycling, this process transforms a random mixture into something not necessarily present at the start but needed at the moment of recycling. We have recently shown that the process of protein recycling can be performed *in vitro* and called it NaCRe (Nature Inspired Circular Recycling). In a previous NaCRe proof-of-concept experiment, we started with various protein mixtures but were able to produce only small quantities of recycled protein, in the microgram scale. Here, we show that NaCRe can be used to convert milligrams of a protein mixture containing one of the most common protein materials (silk) into a milligram of an hydrogel made of green fluorescent protein (GFP). We show that in order for NaCRe to be efficient the starting protein mixture must contain a good balance of all AAs and discuss the challenges encountered when scaling up NaCRe.

## Abstract 2

Protein metabolism is a wonderful example of polymer recycling. A random mixture of proteins gets digested into amino acids, which then can be used by cells to produce whichever protein is needed at the time of synthesis.

## Graphical abstract

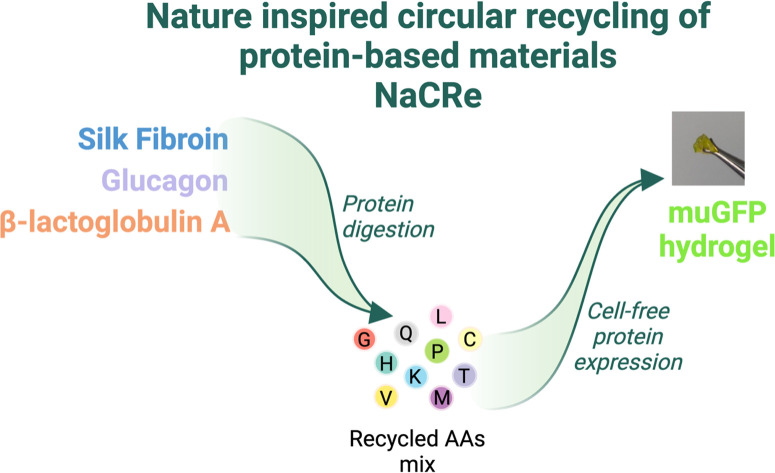

**Sustainability spotlight**

In 2070, the world will be inhabited by 11 billion people, and close to 600 million tons of plastic could be produced yearly. Even if all polymers were to be bio-sourced and bio-degradable, there would still be a huge sustainability challenge in terms of both sourcing and disposal. Humanity has to move towards the principles of a circular economy, where materials, once produced, remain in usage for the longest possible amount of time, taxing Earth the minimum possible. In this work, we propose a strategy to achieve circular recycling of protein-based materials, a novel class of biobased polymeric materials with great potential to substitute fossil-based plastics. This research aligns with the UN's SDG of responsible production and climate action.

## Introduction

Protein-based materials are a developing class of bio-based polymeric materials with considerable potential for replacing some fossil-based materials. With the current climate crisis and the increasing demand for materials production, it is reasonable to direct efforts towards developing protein-based materials, especially when these proteins can be derived from food and agricultural waste. Existing proteins such as silk fibroin have already shown excellent performance as bioplastics,^1^ electronic devices,^2^ implants,^3^ and on-skin electronics^4^ and are still under further development.^5^ In addition, food waste proteins such as whey, soy, lysozyme, β-lactoglobulin and other plant proteins are being studied for the development of CO_2_ capture devices,^6^ water purification devices,^7^ as well as bioplastics.^8^ At the same time, protein engineering is enabling us to understand better the properties of proteins and protein materials. Naturally occurring protein materials serve as an inspiration and as starting points for the development of novel materials with desired properties, expanding their use towards applications such as shock absorbing materials,^9^ elastomers,^10,11^ adhesives,^12^ viscoelastic materials for 3D printing^13^ and hydrogel fibers.^14,15^

We can also get inspiration from Nature when thinking about recycling protein-based materials. Proteins can be cleaved at the amide bond, leading to a pool of amino acid (AA) monomers, in a process that in biology is called protein catabolism. These AAs can be used to build new proteins on demand. Due to the sequence-defined nature of proteins, the newly produced protein can have completely different properties and functions from the initial one. This feature is key for living systems to be able to achieve a broad range of functions that proteins must have. In summary, one approach to recycle proteins in nature is to start with a random protein mixture that gets digested into a pool of AAs, which are then used by ribosomes to produce a totally new protein that typically has nothing in common with the starting ones.

**Fig. 1:**
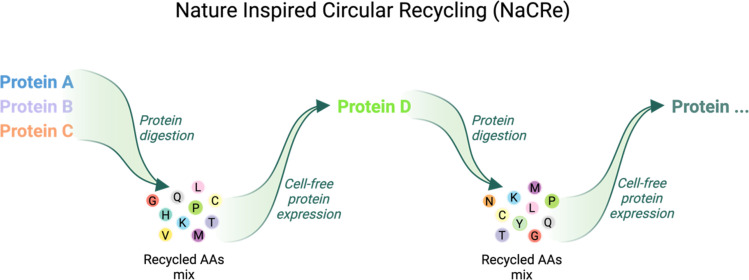
Schematic representation of NaCRe for protein-based materials.

We showed that this recycling can be reproduced in a laboratory environment *in vitro* and called this process Nature inspired circular recycling (NaCRe)^16^ (Fig. 1). In our previous work, a mixture of three proteins was incubated in a two-step *in vitro* digestion reaction, starting with a protease and followed by an aminopeptidase. This produced a mixture of AAs, that, in turn, was introduced into a reconstituted *in vitro* protein synthesis system lacking AAs to produce a different protein than the proteins that constituted the starting mixture. The recycled protein was functional, and could also be degraded to produce AAs, that could be used again to produce a third functional protein, different from any of the previous ones, illustrating the circularity of the building blocks. The circularity of building blocks within different sequence-defined polymers should hold true as well for any other class of sequence-defined polymers, such as nucleic acids. Indeed, we have shown that nucleotides can be recycled *in vitro* in a circular way as well.^17^ Specifically, linear DNA templates were synthesized entirely with nucleotides recovered from calf-DNA.^17^

Previous work showed that these natural sequence-defined polymers can be depolymerized, and their building blocks can be used to produce new biopolymers on demand. Yet, the previous proof-of-concept example was performed on a very small scale. In this work, we aim to show that NaCRe can be used to recycle protein-based materials. We took silk fibroin protein and mixed it with β-lactoglobulin A (a protein present in cow milk) and glucagon (a synthetic peptide) to complement the AA composition. We degraded this mixture into a pool of AAs that we consequently used as the AA source in an *E. coli* cell extract to produce muGFP (monomeric ultra-stable Green Fluorescent Protein), which was purified by affinity chromatography and assembled into a cross-linked hydrogel, by means of 1-(3-dimethylaminopropyl)-3-ethylcarbodiimide hydrochloride (EDC)/*N*-hydroxysuccinimide (NHS) coupling. Furthermore, this recycled hydrogel was again degraded to AAs using NaCRe, confirming the circularity of protein-based materials.

## Results and discussion

**Fig. 2:**
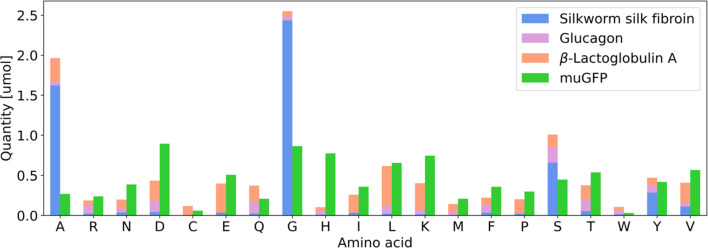
Bar plot of the theoretical quantity in μmol (*y* axis) of each of the 20 AAs (*x* axis) contained in 1 mg of a protein mix composed of 0.416 mg of silk fibroin, 0.416 mg of β-lactoglobulin A, and 0.166 mg of glucagon (in blue, orange and violet, respectively) and in 1 mg of muGFP (in green).

The goal of this work was to use NaCRe to transform one protein-based material into another at a scale that would allow purification of the protein product and its transformation into a material. We set out to produce a muGFP-based hydrogel. We chose muGFP because it expresses well in cell-free transcription–translation systems, and we chose to form a hydrogel because recently a general method to produce protein-based hydrogels was shown using bovine serum albumin (BSA).^18^ muGFP's amino acid composition is shown in Fig. 2. We wanted to start NaCRe from a commonly used protein material and therefore chose silk fibroin, whose AA composition is shown in Fig. 2 in blue. It is immediately evident that silk fibroin alone cannot be used to produce muGFP as, for example, it almost completely lacks leucine. Nevertheless, being a technologically relevant and very abundant protein, we believe it is significant to show that its circular recycling is possible through NaCRe. To compensate for this shortcoming and also to show NaCRe's versatility, we decided to start from a mixture of silk fibroin with glucagon and β-lactoglobulin A. The latter is a protein obtained as a side product of cheese production and has been used to produce water filters.^19^

Typically, we think of NaCRe as a two-step process, “digestion”, *i.e.* the depolymerization of proteins into their constituent AAs, and “synthesis”, *i.e.* the polymerization of the recycled AAs into a new protein. In reality, the first step of NaCRe is protein extraction and solubilization from their starting material or source. Neither in this nor in our previous paper, we have performed this step, and we have always relied on commercial vendors and on collaborators to achieve suitable protein solutions.

In order to scale up NaCRe, one needs to produce large quantities of AAs in the “digestion” step. The recovered free AAs need to be introduced at an optimal concentration in the cell-free expression system (approximately 1 mM of each AA) for efficient conversion into protein (Fig. S1†) during the synthesis step of NaCRe. Thus, to scale-up the digestion step of NaCRe, we applied a protocol based on the previously published one,^16^ with some noticeable differences. Specifically, the volume used was changed from 500 μL to 10 mL, and the starting concentration was increased from 1 mg mL^−1^ to 5 mg mL^−1^. The results of the digestion are detailed below.

**Fig. 3:**
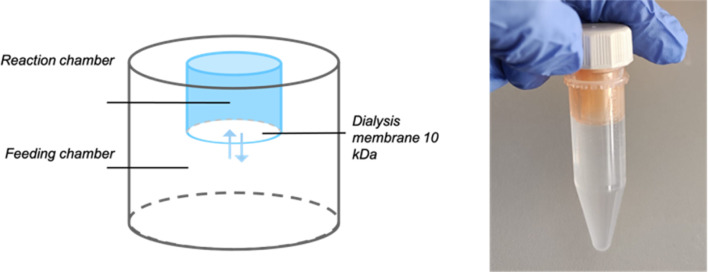
Schematic drawing and a photograph of the real set-up for cell-free protein expression reactions supplied through a dialysis chamber.

In NaCRe, the most challenging step to scale up is the protein expression. Cell-free expression technology shows great potential for NaCRe as many types of proteins can be expressed. Importantly, in these systems, one can ensure that the only free AAs present are the recycled ones, because AAs are supplied to the system. It is thus possible to prove that the expressed proteins are mainly composed of recycled AAs (Fig. S2†). We moved away from reconstituted protein synthesis systems (previously we used commercial PURE systems (Protein synthesis Using Recombinant Elements)) because of their high price when commercially obtained. *E. coli* cell extracts are the most efficient systems in terms of preparation effort, costs and yield, when no post-translational modifications are required on the final product.^20^ Cell-free systems can in turn be enhanced using a dialysis chamber containing more AAs, nucleotides, and energy resources. Dialysis was shown to increase protein yields up to approximately 1 mg mL^−1^ through the extension of the expression reaction due to the continuous supply of AAs and energy molecules and removal of inhibitory by-products.^21,22^*E. coli* lysates can be produced in the lab, but we chose to work with a commercial source to ensure reproducibility of the process. We chose the RTS *E. coli* HY systems from biotechrabbit GmbH, because they provide the lysate, the reaction mix and the AAs independently. This allowed us to substitute the AA mixture with our recycled AA mixture. The reactions were assembled in 10 kDa cut-off dialysis cups, which were inserted in 5 mL Eppendorf tubes with screw caps, as illustrated in Fig. 3. We worked with the Slide-A-Lyzer 2 mL dialysis cups for reaction volumes of 500 μL, supplied by 4.5 mL of feeding mix, so as to have a roughly 10 times larger volume in the dialysis chamber with respect to the reaction.

**Fig. 4:**
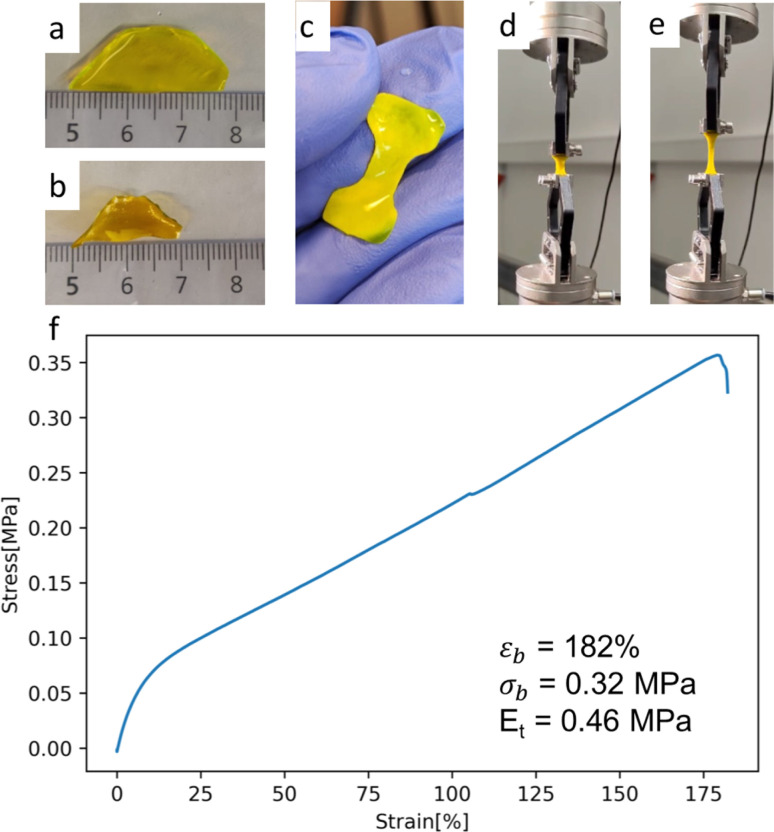
(a) and (b) Pictures of the swelling test of the prototype muGFP hydrogel film. (c) Picture of a dog-bone cut from a prototype muGFP hydrogel film (0.4 mm thick and 5.2 mm thin). (d) and (e) Pictures of the muGFP hydrogel prototype under tensile testing. (f) Stress–strain plot obtained from tensile testing of the prototype muGFP hydrogel.

We chose to express a His-tagged version of muGFP for ease of purification; the purified protein was then cross-linked with EDC–NHS according to an approach recently developed for BSA.^18^ The resulting material is a yellow and fluorescent hydrogel (Fig. S3†). We first expressed muGFP in *E. coli* cells and used it to characterize the mechanical properties of the resulting gel. Briefly, the plasmid was transformed into BL21(DE3) cells. 12 L of AutoTB media was grown at 37 °C until OD >0.8 and switched to 18 °C overnight. Following that, the cells were pelleted and lysed by sonication. Purification was performed through FPLC with a NiNTA column, followed by dialysis against MilliQ water. Around 1.6 g muGFP was obtained, which was then lyophilized and stored at 4 °C. The protein powder was then used to test the muGFP hydrogel formulation. 2 mL of 100 mg mL^−1^ muGFP in MilliQ water was prepared and drop cast onto a polystyrene surface. Before the solution could dry, NHS and EDC dissolved at 1 g mL^−1^ were added sequentially, each at 9% weight ratio, and mixed with the help of a 1 mL pipette. The mix was left to dry at room temperature overnight. Rehydration in MilliQ water led to a hydrogel film, whose swelling ratio could be measured by calculating the weight ratio of the wet sample over the dry sample. The process was repeated three times. The hydrogel film could be cut into a dog-bone shape for tensile testing (Fig. 4). The hydrogel had a Young's modulus of *E*_t_ = 0.46 MPa with an ultimate tensile strength of *σ*_b_ = 0.32 MPa. Both values are very similar to what was reported for the BSA-based hydrogel (for the case where the protein was not denatured and cross-linked), suggesting that similar cross-linking to the one previously reported also occurred in the case of muGFP. The main difference is that for the muGFP hydrogel we find a maximum elongation of 182% that is close to five times smaller to what was reported for BSA (538%). This difference is probably due to a different degree of cross-linking or intrinsic differences between the proteins, such as the molecular weights (BSA 66.3 kDa, as opposed to muGFP that is 34 kDa). The swelling ratio obtained was 2.3, confirming the cross-linking of the proteins.

We then expressed muGFP from a *E. coli* lysate using commercial amino acids. Briefly, 2 mL cell-free expression reactions were assembled with the muGFP plasmid template and a 0.5 mL aliquot was placed in the dialysis cup that was inserted into the dialysis outer chamber and that contained the corresponding amount of feeding solution. The reactions were assembled as specified by the vendor's user manual and incubated at 32 °C, shaking at 800 rpm in a thermoblock for 24 h. After incubation, the reaction mixture was transferred into a 2 mL tube and centrifuged for 15 minutes at 21 000 RCF. The supernatant was run through an affinity column with NiNTA beads following standard His-tag purification procedures, as specified in the ESI Methods section 8.5,† and eluted in imidazole buffer. Lastly, it was dialyzed against MilliQ water and freeze dried. About 0.5 mg of yellow powder was recovered, which was then assembled into a hydrogel according to the procedure already described. The resulting material was found to be insoluble in water over more than three weeks (Fig. S4 and S5†).

**Fig. 5:**
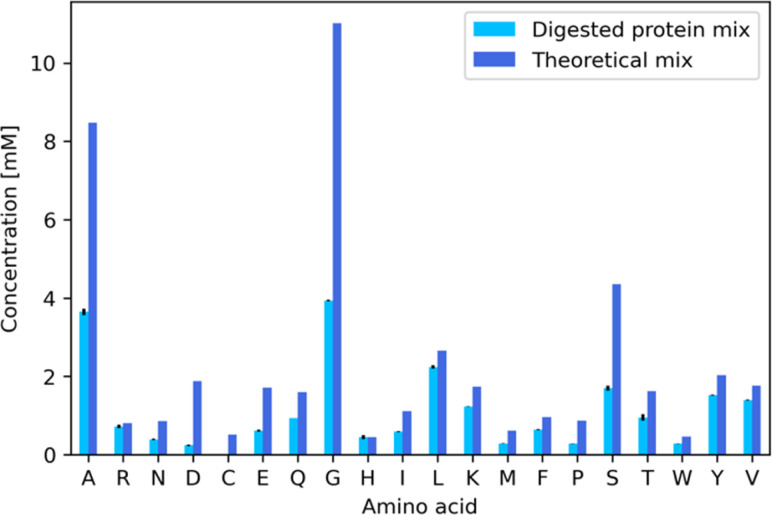
Bar plot of the concentration of each AA in the digestion product of silk fibroin, β-lactoglobulin A and glucagon. Light blue represents the HPLC-MSMS quantification result of the digestion product. Dark blue represents the theoretical concentration, for comparison.

We then used AAs recovered through the digestion of glucagon, silk fibroin and β-lactoglobulin A to produce a recycled muGFP hydrogel. The digestion contained a total protein concentration of 5 mg mL^−1^, and it was composed of the three aforementioned proteins mixed at a ratio of 1/6 : 5/12 : 5/12, respectively. This concentration was chosen because it results in AAs at concentrations ranging from 0.5 mM to 8 mM, which in turn lead to a more efficient protein expression than in the previously developed protocol^16^ (Fig. S6†). The lower amount of glucagon with respect to the other two proteins was chosen to improve the solubility of the mixture, as glucagon was not soluble otherwise. 10 mL of the protein mixture was digested in a two-step enzymatic hydrolysis. First, proteins underwent a cleavage step by incubation with thermolysin protease at a 1 : 20 weight ratio of enzyme to protein. Second, the cleavage product was digested by incubating with leucine aminopeptidase (LAP) at a 1 : 25 weight ratio. Digestion products were filtered with a 3 kDa cut-off centrifugal filter to remove the enzymes as well as undigested proteins. The filtrates were analyzed using a HPLC coupled with a triple quadrupole mass spectrometry analyser (HPLC-MSMS). In Fig. 5 as well as in Table 1, we show the amounts of AAs obtained after digestion and we compare them with the theoretical amounts. It is immediately evident that the yield of digestion changed from one AA to another, ranging from 94% for histidine to 29% of proline. The large differences in part are due to the different sources of these AAs, for example, histidine comes mainly from β-lactoglobulin A and glucagon, while proline comes almost exclusively from β-lactoglobulin A. The three AAs that mainly come from silk fibroin (alanine, glycine and serine) have a similar yield at ∼38%. We obtained an average AA concentration of 1 mM. Many factors play a role here, for example the protein initial solubility as well as all of the digestion parameters. We are currently working to improve the yields of digestion by the development of a robotic platform that will allow us to produce a number of experiments that vary all of the many parameters involved in the digestion. Further screening could consist of searching for an alternative enzyme cocktail that could digest mixtures of proteins robustly.

**Table 1:** HPLC-MSMS quantification results of the digestion product silk fibroin, β-lactoglobulin A and glucagon

Amino acid	Concentration [mM]			% *E*/*T*^b^
	Experiment (*E*)	Theory (*T*)	Negative control^a^	
Alanine (A)	3.64 ± 0.08	8.48	0.05	42.31 ± 0.94
Arginine (R)	0.72 ± 0.04	0.81	0.04	85.17 ± 5.21
Asparagine (N)	0.38 ± 0.02	0.86	0.03	41.02 ± 256
Aspartic acid (D)	0.024 ±0.01	1.88	0.02	12.79 ± 0.53
Cysteine (C)^c^	NAN	0.51	NAN	NAN
Glutamic acid (E)	0.61 ± 0.03	1.72	0.02	34.27 ± 1.58
Glutamine (Q)	0.83 ± 0.00	1.60	0.03	50.35 ± 0.06
Glycine (G)	3.93 ± 0.02	11.01	0.04	35.33 ± 0.16
Histidine (H)	0.45 ± 0.06	0.45	0.02	94.91 ± 12.54
Isoleucine (I)	0.60 ± 0.01	1.12	0.03	50.47 ± 1.18
Leucine (L)	2.24 ± 0.05	2.66	0.07	81.67 ± 1.77
Lysine (K)	1.23 ± 0.01	1.74	0.03	68.92 ± 0.51
Methionine (M)	0.29 ± 0.01	0.62	0.00	46.30 ± 1.18
Phenylalanine (F)	0.64 ± 0.01	0.96	0.03	63.76 ± 0.97
Proline (P)	0.28 ± 0.00	0.87	0.03	28.86 ± 0.23
Serine (S)	1.70 ± 0.06	4.35	0.05	37.94 ± 1.41
Threonine (T)	0.95 ± 0.08	1.63	0.04	56.03 ± 4.84
Tryptophan (W)	0.28 ± 0.00	0.46	0.01	58.37± 0.34
Tyrosine (Y)	1.54 ± 0.01	2.03	0.05	73.40 ± 0.41
Valine (V)	1.40 ± 0.01	1.76	0.05	76.21 ± 0.80

a Incubation of the enzymes alone.

b Percentage of the experimental values with respect to the theoretical ones, with the subtraction of the negative control. This is a measure of the percentage of recovery of amino acids from the protein mix that we aim to recycle.

c Cysteine has very low sensitivity for reliable quantification with the rest of the amino acids.

**Fig. 6:**
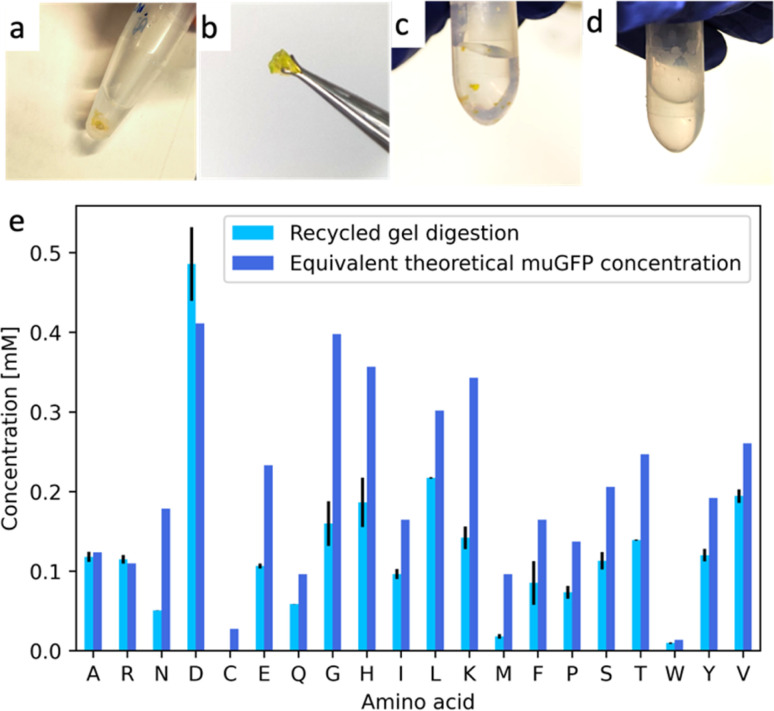
(a) and (b) Pictures of the muGFP-based hydrogel cross-linked through EDC–NHS coupling, made out of recycled AAs from silk fibroin, β-lactoglobulin A and glucagon. (c) Picture of the manually ground recycled hydrogel resuspended in buffer prior to digestion. (d) Picture of the recycled hydrogel after cleavage incubation. No pellet can be distinguished. (e) Bar plot of the concentration of each AA in the digestion product of the muGFP recycled hydrogel. Light blue represents the HPLC-MSMS quantification result of the digestion product. Dark blue represents the theoretical concentration from the corresponding amount of muGFP, for comparison.

A total of 3 mL of cell-free expression reactions were assembled by mixing the lysate and the reaction mix, as specified by the vendor but without mixing the provided AA solution. Instead, we added 0.9 mL of the digested AA solution. 9 mL of the same digested AA solution was also introduced in the corresponding feeding mix. The reactions produced fluorescent muGFP synthesized from recycled AAs (Fig. S7†). Purification of the soluble fraction of the expression reaction, dialysis, and freeze drying were performed as above. We achieved ∼1 mg of recycled muGFP (Fig. S8†). To quantify the yield of this product, one must first determine the limiting AA,^16^ which in our case was histidine. With 0.62 mg of starting histidine in the mixture, a complete conversion could give rise to 5.2 mg of muGFP, hence the yield of NaCRe that we achieved was of ∼20%. We cross-linked the recycled muGFP with EDC–NHS as before. We obtained a yellow-coloured hydrogel shown in Fig. 6a and b. The water insolubility of the product shows that the recycled protein was successfully cross-linked, probably in a similar way to the hydrogel shown in Fig. 4. It is important to notice that the produced muGFP hydrogel possesses properties that differ significantly from the ones of the starting silk fibroin as well as those of β-lactoglobulin A.

One of the key concepts of NaCRe is that it is a circular process for recycling proteins (and specifically protein-based materials) where AAs can be recovered with minimal loss. To better substantiate this point, we showed that the muGFP hydrogels could be readily decomposed into intact AAs. After manual grinding, the recycled hydrogel was digested using the NaCRe protocol. The first step of digestion is an enzymatic process at 85 °C. We performed a control experiment keeping a control ground hydrogel at such temperature in the absence of enzyme to show that no loss of mechanical integrity occurred (Fig. S9†). In Fig. 6e and Table 2, we show the mass spectrometry detection and quantification of the AAs recovered from the process. The yields vary from AA to AA ranging from 19% (methionine) to 100% for aspartic and glutamic acid. In Table 2, one can see that the yield exceeds 100% for some AA; this happens because some enzymes digest themselves. It is important to note that protein-based hydrogels will only be fully circularly recyclable through this process if they have been designed “NaCRe compatible”, meaning the cross-linking strategy is such that it can be cleaved by a non-specific protease. Else, a pre-solubilization step would be required in order to release the protein and recycle the AAs of such material.

**Table 2:** HPLC-MSMS quantification results of the digestion product of the recycled muGFP hydrogel

Amino acid	Concentration [mM]		**%** *E*/*T*^a^
	Experiment (*E*)	Theory (*T*)	
Alanine (A)	0.12 ± 0.01	0.12	95.58 ± 5.15
Arginine (R)	0.11 ± 0.01	0.11	104.76 ± 4.97
Asparagine (N)	0.05 ± 0.00	0.18	28.50 ± 0.15
Aspartic acid (D)	0.47 ± 0.05	0.41	11.437 ± 12.16
Cysteine (C)^b^	NAN	0.03	NAN
Glutamic acid (E)	0.11 ± 0.00	0.23	45.70 ± 1.31
Glutamine (Q)	0.06 ± 0.00	0.10	61.02 ± 0.14
Glycine (G)	0.16 ± 0.03	0.40	40.19 ± 7.07
Histidine (H)	0.19 ± 0.03	0.36	52.30 ± 8.69
Isoleucine (I)	0.10 ± 0.01	0.16	58.59 ± 3.81
Leucine (L)	0.22 ± 0.00	0.30	71.99 ± 0.29
Lysine (K)	0.14 ± 0.01	0.34	41.42 ±4.13
Methionine (M)	0.02 ± 0.00	0.10	18.89 ± 3.17
Phenylalanine (F)	0.09 ± 0.03	0.16	51.87 ± 16.68
Proline (P)	0.07 ± 0.01	0.14	53.58 ± 5.89
Serine (S)	0.11 ± 0.01	0.21	55.01 ±5.38
Threonine (T)	0.14 ± 0.00	0.25	56.35 ± 0.29
Tryptophan (W)	0.01 ± 0.00	0.01	71.03 ± 6.52
Tyrosine (Y)	0.12± 0.01	0.19	62.61 ± 4.15
Valine (V)	0.19 ± 0 01	0.26	74.59 ± 3.23

a Percentage of the experimental values with respect to the theoretical ones, with the subtraction of the negative control. This is a measure of the percentage of recovery of amino acids from the protein mix that we aim to recycle.

b Cysteine has very low sensitivity for reliable quantification with the rest of the amino acids.

## Conclusion

In conclusion, we have presented here an example of NaCRe where two proteins commonly used as materials (silk fibroin and β-lactoglobulin A) mixed with one hormone (glucagon) were recycled together to produce a fourth protein (muGFP) that was in turn cross-linked to produce a hydrogel. The recycled material has properties that were distinctively different from those of silk fibroin or of films of β-lactoglobulin A (typically used as water filters). The cross-linking strategy used, being generally applicable to any protein, shows that a broad range of protein materials could be produced with recycled AAs. Additionally, we have also shown that intact AAs can be recovered again from the hydrogel we produced from recycled proteins, and these AAs are available for the expression of future proteins on demand. The main challenges we had were all in the scale up of NaCRe so as to produce larger quantities. This entailed increasing the digestion volume from 500 μL to 10 mL, together with increasing the protein mixture concentration from 1 to 5 mg mL^−1^. In addition, we switched from protein expression in PURE to protein expression in cell lysate complemented with a dialysis chamber with additional energy sources and building blocks. This led to an increase in protein production from ∼1 μg to 1 mg, reducing the laboratory costs from approximately 19 000 € to 1500 € per mg of purified recycled protein. This result is significant because it shows that NaCRe can convert protein materials into totally different protein-based materials. At present, the overall yield of the process is still low, and current efforts are focused on improving it.

## Data availability

Data availability statement for “Nature-Inspired Recycling of a Protein Mixture into a Green Fluorescent Protein-based hydrogel” and all data sets for this paper can be found in Zenodo with doi: https://doi.org/10.5281/zenodo.12700532.

## Author contributions

L. R. J. was responsible for investigation, data curation, and formal analysis. L. R. J. and F. S. conceptualized and wrote the original draft of the manuscript. F. S. and S. M. were responsible for supervision and editing of the final version of the manuscript.

## Conflicts of interest

The authors have no conflict of interest with the content of this paper.

## Supplementary Material

SU-002-D4SU00212A-s001
