# Supplementary material for: Nature-inspired recycling of a protein mixture into a green fluorescent protein-based hydrogel
Source: RSC Sustain. 2024 Jul 23;2(10):2903–9. doi: 10.1039/d4su00212a (PMC11447593; doi:10.1039/d4su00212a)
Supplement: SU-002-D4SU00212A-s001 [file SU-002-D4SU00212A-s001.pdf]

Supplementary Information (SI) for RSC Sustainability.
This journal is © The Royal Society of Chemistry 2024

Supplementary Information - Nature-Inspired Recycling of a Protein Mixture into a Green Fluorescent Protein-based hydrogel

Laura Roset Julià[1,2], Sebastian J. Maerkl[3], Francesco Stellacci*[1,2,3,4]

[1]Institute of Materials, Ecole Polytechnique Fédérale de Lausanne (EPFL), 1015 Lausanne, Switzerland

[2]NCCR Bio-Inspired Materials, Ecole Polytechnique Fédérale de Lausanne (EPFL), 1015 Lausanne, Switzerland

[3]Bioengineering Institute, Ecole Polytechnique Fédérale de Lausanne (EPFL), 1015 Lausanne, Switzerland

[4]Global Health Institute, Ecole Polytechnique Fédérale de Lausanne (EPFL), 1015 Lausanne, Switzerland

*Corresponding author: francesco.stellacci@epfl.ch

Supplementary Data

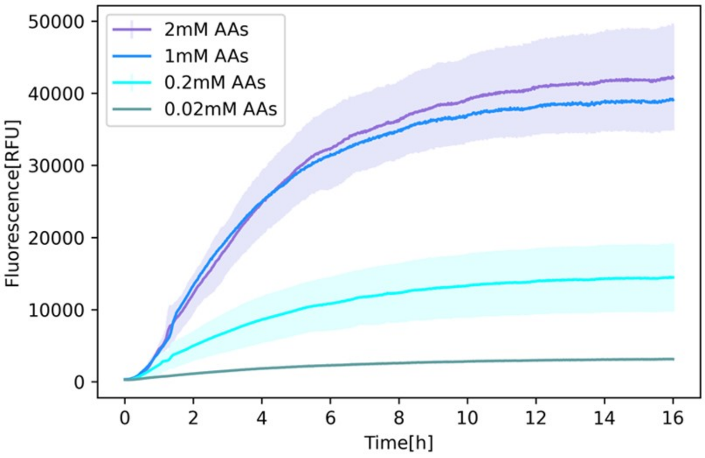

Figure S1: Expression level in cell-free batch reaction as a function of initial AAs concentration. The production yield is determined by the fluorescence of expressed GFP. Shaded areas correspond to the standard deviation from duplicates of the experiment.

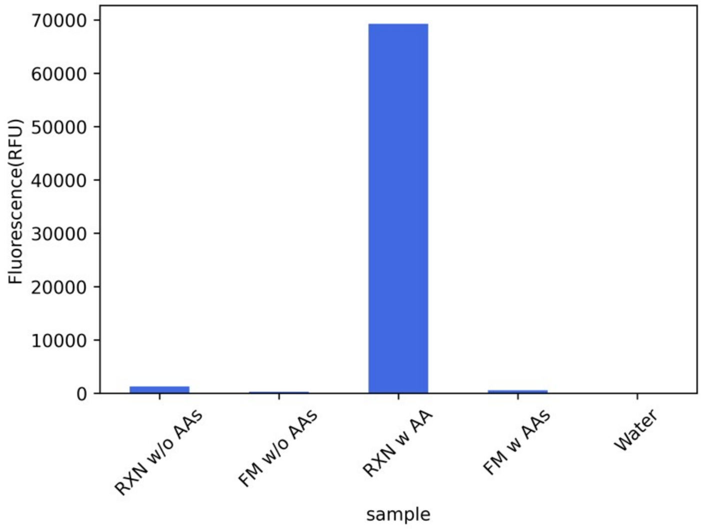

Figure S2: Fluorescence measurement of crude cell-free expression reactions (RXN) with its respective feeding mix (FM) after 24h incubation introducing AAs to the system vs not introducing AAs. Significantly larger fluorescence signal is detected with respect to a control cell-free expression where no AAs were introduced. The feeding chambers (dialysis chambers) are also measured to ensure no product protein is significantly leaking.

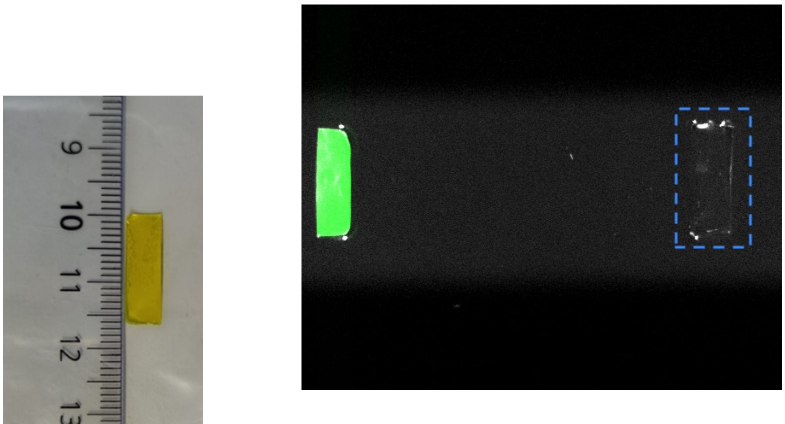

Figure S3: The left figure is a photograph of EDC/NHS cross-linked muGFP hydrogel. The right figure is a fluorescence and bright field image superimposed of EDC/NHS cross-linked muGFP hydrogel (left) and EDC/NHS cross-linked BSA hydrogel (right, marked by the blue dotted rectangle) as a control. Fluorescence imaging (Excitation wavelength: 488 nm) is overlayed with a bright field image for better visualization of the two hydrogels. The muGFP hydrogel saturates the fluorescence channel, while the control hydrogel does not show any signal, showing that the muGFP in the hydrogel is fluorescent.

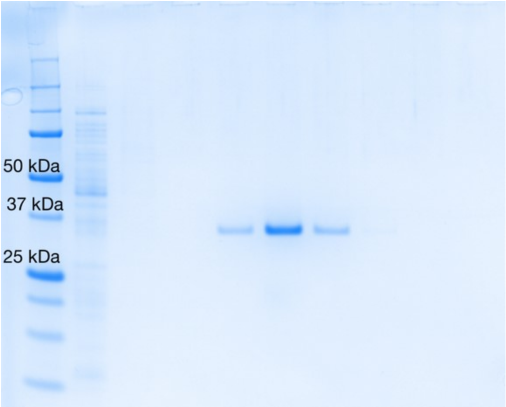

Figure S4: muGFP elution profile of the His-tag purification process from the cell-free expression reaction. Mw of muGFP is 34kDa.

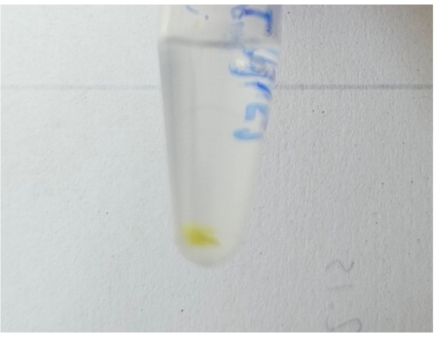

Figure S5: muGFP cross-linked hydrogel from cell-free expressed protein.

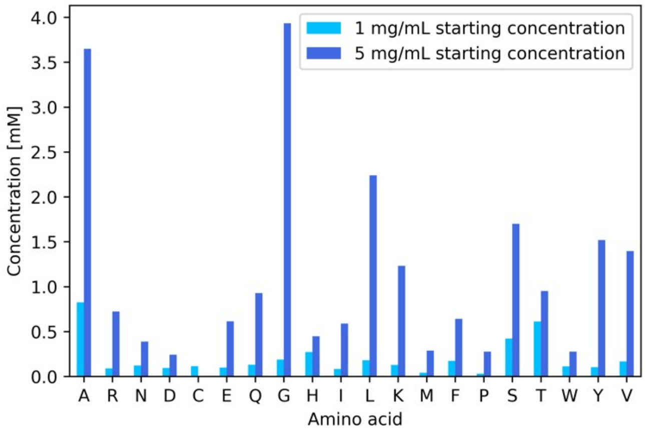

Figure S6: HPLC-MSMS quantification of the initial digestion protocol compared to the one developed for this experiment, with the goal to obtain more concentrated amino acid solutions.

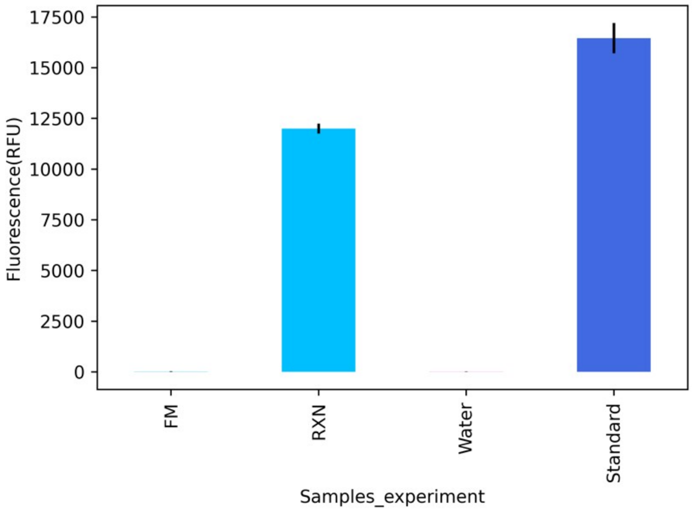

Figure S7: Fluorescence measurement of crude cell-free expression reaction (RXN) with its respective feeding mix (FM) supplied with recycled amino acids. Fluorescence indicates the successful production of functional recycled muGFP. The last column is a control of 0.467 mg/mL muGFP in MilliQ.

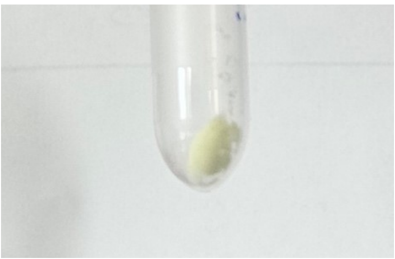

Figure S8: Lyophilized recycled muGFP

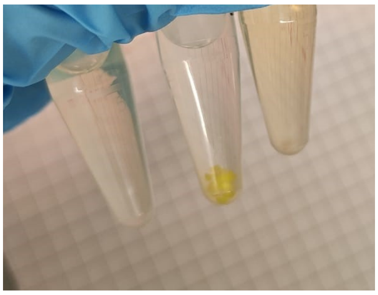

Figure S9: Cleavage reaction product of a muGFP hydrogel cross-linked with 18% EDC NHS. From left to right: Protein solution + Thermolysin, Cross-linked hydrogel, Cross-linked hydrogel + Thermolysin

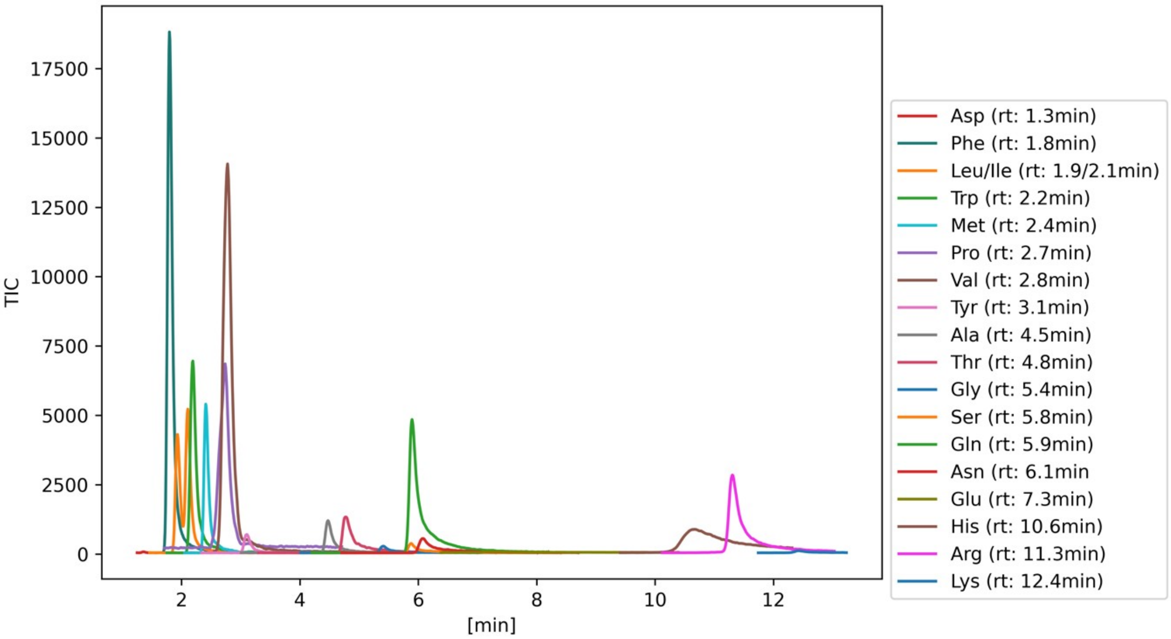

Figure S10: Chromatogram acquired through DMRM with an HPLC-QQQ (as detailed in Experimental Section 7) of the first point of the calibration curve (mixture of the 20 AAs, each at 0.5 μg/mL). Each identified amino acid is plotted with a different color for better visualization, and its retention time is specified in the legend.

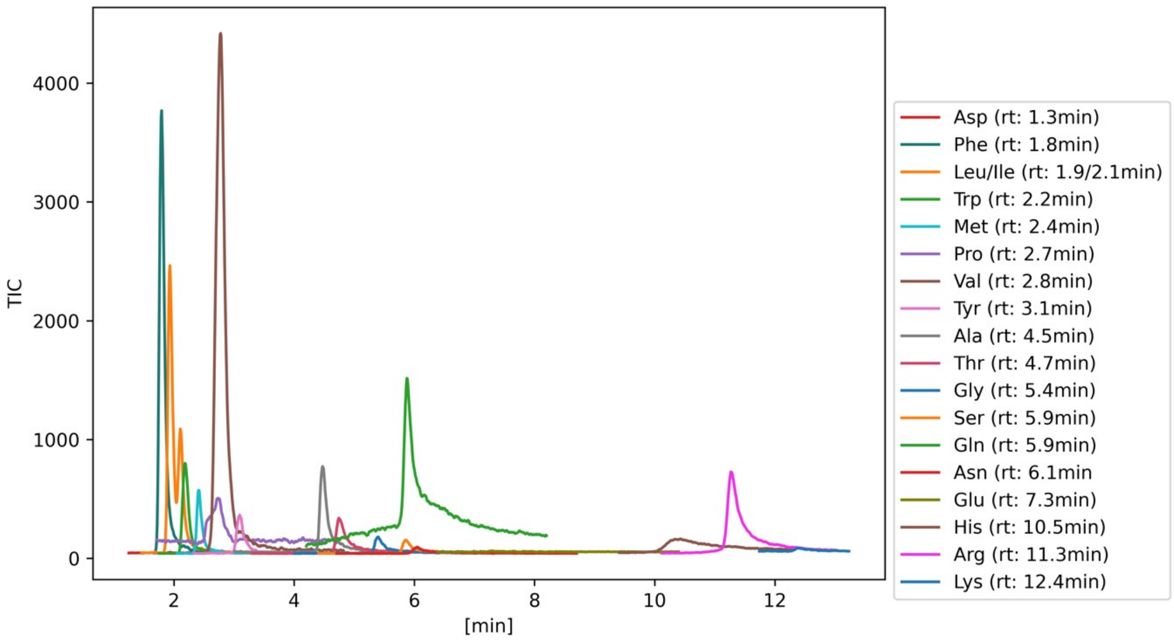

Figure S11: Chromatogram acquired through DMRM with an HPLC-QQQ (as detailed in Experimental Section 7) of the digestion product of the mixture of silk fibroin, β-lactoglobulin A and glucagon, diluted x1000. Each identified amino acid is plotted with a different color for better visualization, and its retention time is specified in the legend.

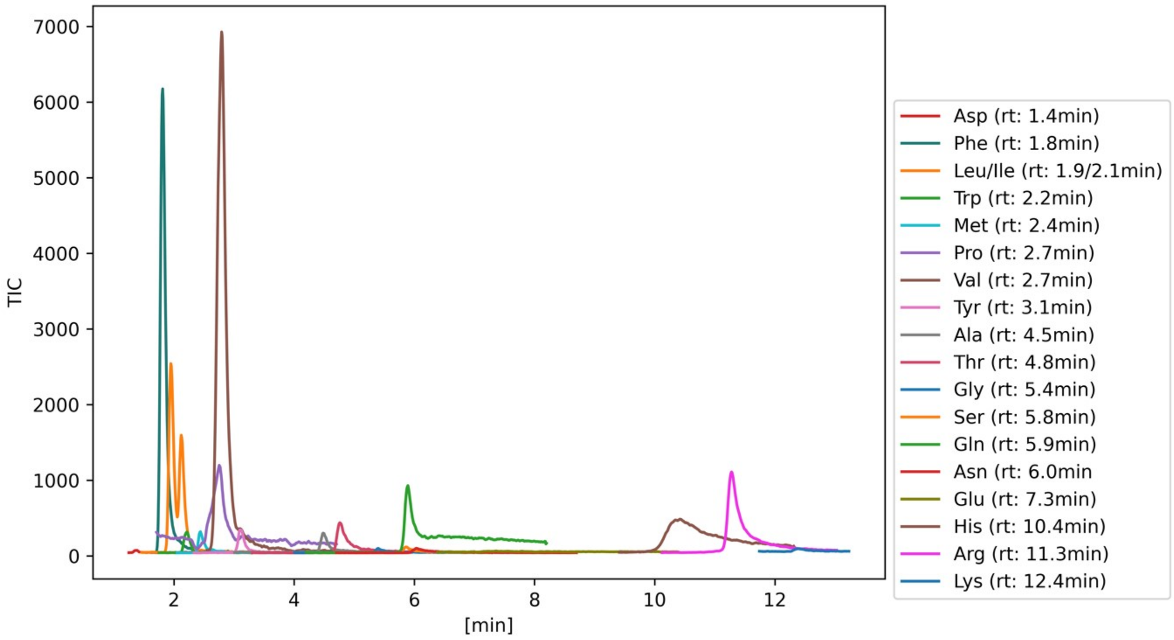

Figure S12: Chromatogram acquired through DMRM with an HPLC-QQQ (as detailed in Experimental Section 7) of the digestion product of the recycled muGFP hydrogel x100 dilution. Each identified amino acid is plotted with a different color for better visualization, and its retention time is specified in the legend.

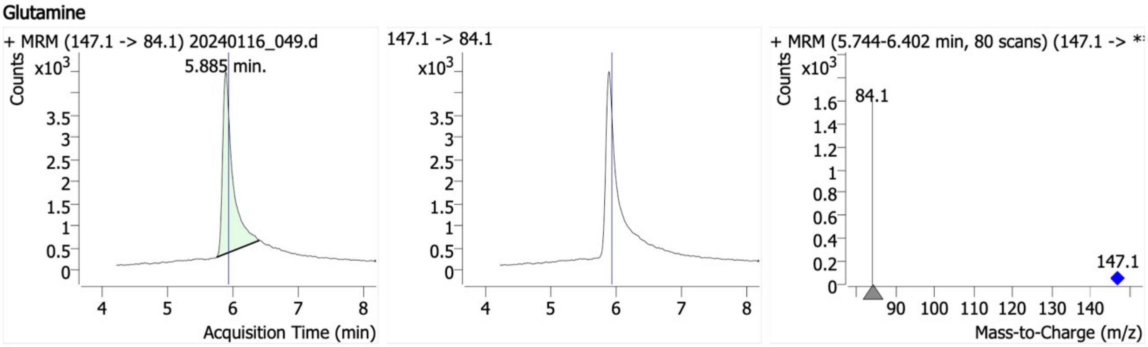

Figure S13: Example of a chromatogram of the MS fragment from glutamine (left and middle), and of an MS spectrum of the fragment generated for glutamine (right). The data presented were generated for the sample whose complete set of chromatograms is shown in Figure S10. The complete data set can be found together with all other data for this manuscript at https://doi.org/10.5281/zenodo.12700532 .

Materials and methods

Reagents

Thermolysin from Geobacillus stearothermophilus (P1512); Leucine Aminopeptidase, microsomal from porcine kidney (L6007-25UN); Glucagon Synthetic (G2044); Silk fibroin solution (5154); β-Lactoglobulin A from bovine milk (L7880); L-Amino Acids (LAA21-1KT); Laemli buffer 2x; Nuclease Free Water (W4502-1L) were purchased from Sigma-Aldrich. Ammonium Formate, Optima™ LC/MS Grade (A11550) was purchased from Fisher Chemical. Formic acid ≥99 for LC-MS (84865.260) was purchased from VWR Chemicals. Acetonitrile, ULC-MS (BSO0001204102BS-PACK) was purchased from Biosolve. Vials for HPLC-MSMS analysis were purchased from Agilent Technologies. SDS Page precast gels (NP0322BOX) were purchased from Thermofisher Scientific; InstantBlue® Coomassie Protein Stain (ISB1L) (ab119211-1000ml) from Abcam; Precision Plus Protein Unstained Protein Standards, Strep-tagged recombinant, 1 ml (1610363) was purchased from Biorad.

Instruments

All plate reader measurements were done in a BioTek Synergy H1 microplate reader. The incubations were done in the Eppendorf Thermomixer with the 15 mL block or the 1.5 mL bock accordingly. For tensile testing we used the AllroundLine Z005, 5 kN load cell (Zwick Roell). Milli-Q was supplied by the Milli-Q Integral 3/5/10/15 System from Merck Millipore. HPLC-MSMS was performed with a 6470B Triple Quadrupole Mass Spectrometer coupled to the 1260 series HPLC system (Agilent Technologies). Fluorescence of the cross-linked muGFP hydrogel prototype was imaged with Ibright 1500 from Invitrogen.

Plasmid sequence:

CGGTGATGCCGGCCACGATGCGTCCGGCGTAGAGGATCGAGATCGATCTCGATCCCGCGAAATTAATACGACTCACTATAGGGGAATT
GTGAGCGGATAACAATTCCCCTCTAGAAATAATTTTGTTTAACTTTAAGAAGGAGATATACATATGAAACATCATCATCACCACCATCATC
ACCACCACCATCACCATCATGGCGCCTCTATGGATTACAAAGACCATGATGGTGATTACAAGGATCATGATATTGATTATAAAGATGATG
ATGATAAAGGTTCCGGGTCTGGTGAAAACCTCTACTTTCAAGGGTCGGGATCCATGGTTTCCAAAGGAGAAGAACTGTTTACCGGTGTT
GTACCAATTCTCGTAGAACTCGATGGAGATGTAAACGGGCATAAATTTTCAGTGCGCGGCGAGGGCGAAGGAGATGCCACAAACGGCA
AACTGACCCTTAAATTTATTTGCACGACCGGCAAATTACCAGTTCCTTGGCCTACGCTGGTCACCACGCTCACCTATGGGGTATTATGCTT
TAGCCGCTATCCGGATCACATGAAACGCCATGATTTCTTTAAAAGTGCTATGCCAGAAGGTTATGTACAGGAACGCACGATTAGCTTTAA
AGATGATGGGACGTATAAAACCCGCGCCGAGGTAAAATTTGAAGGAGATACCTTAGTAAACCGCATTGAACTCAAGGGGATTGATTTT
AAAGAGGACGGTAACATTCTGGGTCATAAACTTGAGTACAACTTTAACTCACACAACGTTTACATTACCGCGGATAAACAGAAGAACGG
TATTAAAGCGTACTTTAAGATTCGCCATAACGTCGAAGATGGCAGTGTTCAGCTGGCCGATCATTATCAGCAGAACACGCCGATTGGCG
ATGGCCCTGTTTTGTTACCGGATAACCATTATTTATCGACTCAGAGCGTCTTAAGTAAAGATCCAAACGAGAAACGCGATCACATGGTTC
TCTTAGAAGATGTTACCGCCGCCGGCATTACACATGGCATGGATGAACTGTATAAATGATAGGCGGCCGCAGGACTGAATGATATTTTC
GAAGCACAAAAGATTGAGTGGCATGAAGCTAGCGAGAATTTGTATTTTCAAGGTAGTGCTTGGTCGCACCCTCAATTCGAAAAGGGCG
GCGGTAGTGGCGGTGGTTCAGGCGGTTCCGCGTGGAGTCACCCGCAATTCGAGAAAGGCGCTTGATAGCTCGAGCACCACCACCACCA
CCACTGAGATCCGGCTGCTAACAAAGCCCGAAAGGAAGCTGAGTTGGCTGCTGCCACCGCTGAGCAATAACTAGCATAACCCCTTGGG
GCCTCTAAACGGGTCTTGAGGGGTTTTTTGCTGAAAGGAGGAACTATATCCGGATTGGCGAATGGGACGCGCCCTGTAGCGGCGCATT
AAGCGCGGCGGGTGTGGTGGTTACGCGCAGCGTGACCGCTACACTTGCCAGCGCCCTAGCGCCCGCTCCTTTCGCTTTCTTCCCTTCCTT
TCTCGCCACGTTCGCCGGCTTTCCCCGTCAAGCTCTAAATCGGGGGCTCCCTTTAGGGTTCCGATTTAGTGCTTTACGGCACCTCGACCCC
AAAAAACTTGATTAGGGTGATGGTTCACGTAGTGGGCCATCGCCCTGATAGACGGTTTTTCGCCCTTTGACGTTGGAGTCCACGTTCTTT
AATAGTGGACTCTTGTTCCAAACTGGAACAACACTCAACCCTATCTCGGTCTATTCTTTTGATTTATAAGGGATTTTGCCGATTTCGGCCT
ATTGGTTAAAAAATGAGCTGATTTAACAAAAATTTAACGCGAATTTTAACAAAATATTAACGCTTACAATTTAGGTGGCACTTTTCGGGG
AAATGTGCGCGGAACCCCTATTTGTTTATTTTTCTAAATACATTCAAATATGTATCCGCTCATGAATTAATTCTTAGAAAAACTCATCGAGC
ATCAAATGAAACTGCAATTTATTCATATCAGGATTATCAATACCATATTTTTGAAAAAGCCGTTTCTGTAATGAAGGAGAAAACTCACCG

AGGCAGTTCCATAGGATGGCAAGATCCTGGTATCGGTCTGCGATTCCGACTCGTCCAACATCAATACAACCTATTAATTTCCCCTCGTCA
AAAATAAGGTTATCAAGTGAGAAATCACCATGAGTGACGACTGAATCCGGTGAGAATGGCAAAAGTTTATGCATTTCTTTCCAGACTTG
TTCAACAGGCCAGCCATTACGCTCGTCATCAAAATCACTCGCATCAACCAAACCGTTATTCATTCGTGATTGCGCCTGAGCGAGACGAAA
TACGCGATCGCTGTTAAAAGGACAATTACAAACAGGAATCGAATGCAACCGGCGCAGGAACACTGCCAGCGCATCAACAATATTTTCAC
CTGAATCAGGATATTCTTCTAATACCTGGAATGCTGTTTTCCCGGGGATCGCAGTGGTGAGTAACCATGCATCATCAGGAGTACGGATA
AAATGCTTGATGGTCGGAAGAGGCATAAATTCCGTCAGCCAGTTTAGTCTGACCATCTCATCTGTAACATCATTGGCAACGCTACCTTTG
CCATGTTTCAGAAACAACTCTGGCGCATCGGGCTTCCCATACAATCGATAGATTGTCGCACCTGATTGCCCGACATTATCGCGAGCCCAT
TTATACCCATATAAATCAGCATCCATGTTGGAATTTAATCGCGGCCTAGAGCAAGACGTTTCCCGTTGAATATGGCTCATAACACCCCTTG
TATTACTGTTTATGTAAGCAGACAGTTTTATTGTTCATGACCAAAATCCCTTAACGTGAGTTTTCGTTCCACTGAGCGTCAGACCCCGTAG
AAAAGATCAAAGGATCTTCTTGAGATCCTTTTTTTCTGCGCGTAATCTGCTGCTTGCAAACAAAAAAACCACCGCTACCAGCGGTGGTTT
GTTTGCCGGATCAAGAGCTACCAACTCTTTTTCCGAAGGTAACTGGCTTCAGCAGAGCGCAGATACCAAATACTGTCCTTCTAGTGTAGC
CGTAGTTAGGCCACCACTTCAAGAACTCTGTAGCACCGCCTACATACCTCGCTCTGCTAATCCTGTTACCAGTGGCTGCTGCCAGTGGCG
ATAAGTCGTGTCTTACCGGGTTGGACTCAAGACGATAGTTACCGGATAAGGCGCAGCGGTCGGGCTGAACGGGGGGTTCGTGCACACA
GCCCAGCTTGGAGCGAACGACCTACACCGAACTGAGATACCTACAGCGTGAGCTATGAGAAAGCGCCACGCTTCCCGAAGGGAGAAAG
GCGGACAGGTATCCGGTAAGCGGCAGGGTCGGAACAGGAGAGCGCACGAGGGAGCTTCCAGGGGGAAACGCCTGGTATCTTTATAGT
CCTGTCGGGTTTCGCCACCTCTGACTTGAGCGTCGATTTTTGTGATGCTCGTCAGGGGGGCGGAGCCTATGGAAAAACGCCAGCAACGC
GGCCTTTTTACGGTTCCTGGCCTTTTGCTGGCCTTTTGCTCACATGTTCTTTCCTGCGTTATCCCCTGATTCTGTGGATAACCGTATTACCG
CCTTTGAGTGAGCTGATACCGCTCGCCGCAGCCGAACGACCGAGCGCAGCGAGTCAGTGAGCGAGGAAGCGGAAGAGCGCCTGATGC
GGTATTTTCTCCTTACGCATCTGTGCGGTATTTCACACCGCAATGGTGCACTCTCAGTACAATCTGCTCTGATGCCGCATAGTTAAGCCAG
TATACACTCCGCTATCGCTACGTGACTGGGTCATGGCTGCGCCCCGACACCCGCCAACACCCGCTGACGCGCCCTGACGGGCTTGTCTGC
TCCCGGCATCCGCTTACAGACAAGCTGTGACCGTCTCCGGGAGCTGCATGTGTCAGAGGTTTTCACCGTCATCACCGAAACGCGCGAGG
CAGCTGCGGTAAAGCTCATCAGCGTGGTCGTGAAGCGATTCACAGATGTCTGCCTGTTCATCCGCGTCCAGCTCGTTGAGTTTCTCCAGA
AGCGTTAATGTCTGGCTTCTGATAAAGCGGGCCATGTTAAGGGCGGTTTTTTCCTGTTTGGTCACTGATGCCTCCGTGTAAGGGGGATTT
CTGTTCATGGGGGTAATGATACCGATGAAACGAGAGAGGATGCTCACGATACGGGTTACTGATGATGAACATGCCCGGTTACTGGAAC
GTTGTGAGGGTAAACAACTGGCGGTATGGATGCGGCGGGACCAGAGAAAAATCACTCAGGGTCAATGCCAGCGCTTCGTTAATACAGA
TGTAGGTGTTCCACAGGGTAGCCAGCAGCATCCTGCGATGCAGATCCGGAACATAATGGTGCAGGGCGCTGACTTCCGCGTTTCCAGAC
TTTACGAAACACGGAAACCGAAGACCATTCATGTTGTTGCTCAGGTCGCAGACGTTTTGCAGCAGCAGTCGCTTCACGTTCGCTCGCGTA
TCGGTGATTCATTCTGCTAACCAGTAAGGCAACCCCGCCAGCCTAGCCGGGTCCTCAACGACAGGAGCACGATCATGCGCACCCGTGGG
GCCGCCATGCCGGCGATAATGGCCTGCTTCTCGCCGAAACGTTTGGTGGCGGGACCAGTGACGAAGGCTTGAGCGAGGGCGTGCAAG
ATTCCGAATACCGCAAGCGACAGGCCGATCATCGTCGCGCTCCAGCGAAAGCGGTCCTCGCCGAAAATGACCCAGAGCGCTGCCGGCA
CCTGTCCTACGAGTTGCATGATAAAGAAGACAGTCATAAGTGCGGCGACGATAGTCATGCCCCGCGCCCACCGGAAGGAGCTGACTGG
GTTGAAGGCTCTCAAGGGCATCGGTCGAGATCCCGGTGCCTAATGAGTGAGCTAACTTACATTAATTGCGTTGCGCTCACTGCCCGCTTT
CCAGTCGGGAAACCTGTCGTGCCAGCTGCATTAATGAATCGGCCAACGCGCGGGGAGAGGCGGTTTGCGTATTGGGCGCCAGGGTGG
TTTTTCTTTTCACCAGTGAGACGGGCAACAGCTGATTGCCCTTCACCGCCTGGCCCTGAGAGAGTTGCAGCAAGCGGTCCACGCTGGTTT
GCCCCAGCAGGCGAAAATCCTGTTTGATGGTGGTTAACGGCGGGATATAACATGAGCTGTCTTCGGTATCGTCGTATCCCACTACCGAG
ATGTCCGCACCAACGCGCAGCCCGGACTCGGTAATGGCGCGCATTGCGCCCAGCGCCATCTGATCGTTGGCAACCAGCATCGCAGTGG
GAACGATGCCCTCATTCAGCATTTGCATGGTTTGTTGAAAACCGGACATGGCACTCCAGTCGCCTTCCCGTTCCGCTATCGGCTGAATTT
GATTGCGAGTGAGATATTTATGCCAGCCAGCCAGACGCAGACGCGCCGAGACAGAACTTAATGGGCCCGCTAACAGCGCGATTTGCTG
GTGACCCAATGCGACCAGATGCTCCACGCCCAGTCGCGTACCGTCTTCATGGGAGAAAATAATACTGTTGATGGGTGTCTGGTCAGAGA
CATCAAGAAATAACGCCGGAACATTAGTGCAGGCAGCTTCCACAGCAATGGCATCCTGGTCATCCAGCGGATAGTTAATGATCAGCCCA
CTGACGCGTTGCGCGAGAAGATTGTGCACCGCCGCTTTACAGGCTTCGACGCCGCTTCGTTCTACCATCGACACCACCACGCTGGCACCC
AGTTGATCGGCGCGAGATTTAATCGCCGCGACAATTTGCGACGGCGCGTGCAGGGCCAGACTGGAGGTGGCAACGCCAATCAGCAAC
GACTGTTTGCCCGCCAGTTGTTGTGCCACGCGGTTGGGAATGTAATTCAGCTCCGCCATCGCCGCTTCCACTTTTTCCCGCGTTTTCGCAG
AAACGTGGCTGGCCTGGTTCACCACGCGGGAAACGGTCTGATAAGAGACACCGGCATACTCTGCGACATCGTATAACGTTACTGGTTTC
ACATTCACCACCCTGAATTGACTCTCTTCCGGGCGCTATCATGCCATACCGCGAAAGGTTTTGCGCCATTCGATGGTGTCCGGGATCTCG
ACGCTCTCCCTTATGCGACTCCTGCATTAGGAAGCAGCCCAGTAGTAGGTTGAGGCCGTTGAGCACCGCCGCCGCAAGGAATGGTGCAT
GCAAGGAGATGGCGCCCAACAGTCCCCCGGCCACGGGGCCTGCCACCATACCCACGCCGAAACAAGCGCTCATGAGCCCGAAGTGGCG
AGCCCGATCTTCCCCATCGGTGATGTCGGCGATATAGGCGCCAGCAACCGCACCTGTGGCG

Protein sequence:
MKHHHHHHHHHHHHHHGASMDYKDHDGDYKDHDIDYKDDDDKGSGSGENLYFQGSGSMVSKGEELFTGVVPILVELDGDVNGHKFSVR
GEGEGDATNGKLTLKFICTTGKLPVPWPTLVTTLTYGVLCFSRYPDHMKRHDFFKSAMPEGYVQERTISFKDDGTYKTRAEVKFEGDTLVNRIE
LKGIDFKEDGNILGHKLEYNFNSHNVYITADKQKNGIKAYFKIRHNVEDGSVQLADHYQQNTPIGDGPVLLPDNHYLSTQSVLSKDPNEKRDH
MVLLEDVTAAGITHGMDELYK*

Experimental details

1. E. Coli expression of muGFP

The plasmid encoding a *E.coli* codon-optimized pet29b-muGFP-2xFlag-10xHis construct was transformed into BL21(DE3) (Lucigen) cells and plated. A steak of cells were grown overnight in LB media supplemented in kanamycin. For every liter of production culture, 20 mL of overnight culture were inoculated into Autoinduction TB (Formedium) media and grown at

37ºC. 12L were grown in total, in batches of 2L in 5 L flash. After 4-5 hours or an OD600 of > 0.8, the incubator temperature was changed to 18ºC. The cultures were grown overnight for at least 18 hours. Cell pellets were harvested and stored at -20ºC. Cell pellets were resuspended in Buffer A (700 mM NaCl, 20 mM HEPES 7.5), supplemented with glycerol to 10% v/v and 10 μL of Turbonuclease, then lysed by sonication. For every 2 L of pellet, approximate volume of lysate was 50 mL. Lysates were clarified by centrifugation, filtered in through a 0.45 um filter and supplemented with 25 mM imidazole. The sample was loaded on to a 25 mL NiNTA column (Cytiva or ProteinArk) and the protein was eluted on a gradient of 5-100% Buffer B (700 mM NaCl, 500 mM imidazole, 20 mM HEPES 7.5) on an AKTA system. Pooled eluted fractions were dialyzed twice in 5 L of MQ water.

2. Preparation of muGFP hydrogel prototype samples

Hydrogel films were prepared as following. 200 mg of muGFP were dissolved in 1.96 mL of MilliQ and were casted in a polystyrene weighting boat. 18 μL of 1g/mL NHS was added, followed by 18 μL of 1 g/mL EDC, and gently mixed with a 1 mL pipet. The mix was let evaporate under the hood over the weekend. This resulted in a brittle film that could be rehydrated into the hydrogel, which was then cut into the desired shape prior to testing.

3. Tensile test

Hydrogel films were cut with a dog-bone cutter for tensile testing, and they were kept for several days in MilliQ water prior to testing, to ensure that swelling equilibrium was reached. The sample had a parallel length of 7.5 mm, a width of 5.2 mm, and a thickness of 0.4 mm. It was clamped in the tensile tester by means of rubber clamps. The test was performed a constant velocity of 20 mm/min. The Young's modulus was calculated as the slope of the initial linear region (from 10% to 15% strain).

4. Swelling test

The swelling test was performed in MilliQ water. A piece of the hydrogel film was let immersed in water to ensure swelling for 3 days. Then, it was weighted after removal of excess water with a lint free tissue, and let dry over 3 more days under the hood, after which it was weighted again. This cycle was performed three times to ensure a stable swelling ratio. Swelling ratio was calculated as the weight of the wet sample divided by the weight of the dry sample.

5. Digestion of proteins

We purchased silk fibroin from a commercial vendor, derived from domesticated *Bombyx Mori* silkworm. The protein solution was most probably produced by a well-established method of first degumming the cocoons to remove sericin and then solubilizing the protein in a LiBr bath[20]. We also purchased β-lactoglobulin A, as opposed to our previous work where we used the fibrillated version of the protein that is used to produce water filters[16]. Glucagon is a hormone; for this paper, we bought the synthetic form. Proteins were reconstituted in digestion buffer, which was composed of Tris−HCl (50 mM), $CaCl_2$ (1 mM), KOH, all at pH 8 except Glucagon that had pH 9.

<u>*5.1 Cleavage reaction:*</u> 10 mL of Cleavage solution was prepared as following: Glucagon, β-Lactoglobulin A and Silk fibroin where mixed in the ratio of (1/6):(5/12):(5/12) respectively, to an overall protein concentration of 4.75 mg/mL in Digestion Buffer. Thermolysin was added to the mixture to a final concentration of 250 μg/mL, to have a 19:1 protein:enzyme mass ratio. The mixture was incubated at 85°C for 6 hours, shaking at 500 rpm. After incubation, samples where filtered with 10 kDa Amicon centrifugal filters (UFC8010) and frozen at -20°C until the next step.

<u>*5.2 Digestion reaction:*</u> Cleaved samples where thawed and Leucine Aminopeptidase was introduced estimating to achieve 25:1 protein:enzyme mass ratio, for an ideal case where all initial protein would be cleaved. Reactions were incubated at 37°C for 8 hours at 500 rpm shaking, followed by 3 kDa amicon centrifugal filtration (UFC8003) and frozen at -20°C. A negative control was prepared with the same concentration of enzymes but no protein to cleave, and followed the same procedure.

6. Digestion of the recycled muGFP hydrogel

The recycled hydrogel was stored dry in an Eppendorf tube sealed with parafilm at 4°C. Prior to digestion, it was manually grinded by putting it between two plastic surfaces and applying pressure, and rehydrated with 473.7 μL of digestion buffer pH 8. After one hour at room temperature, 26.3 μL of Thermolysin solution (1 mg/mL) was added, and the reaction was incubated for 6 hours at 85°C. The result of this reaction is depicted in Figure 6.d, were already no significant pieces of hydrogel can be distinguished. To ensure extensive cleavage, a second cleavage round was performed by adding again the same amount of Thermolysin and letting the incubation take place for 15 hours more. Following, 125 μL of LAP solution (1 mg/mL) were introduced and the reaction was incubated for 8 hours at 37ºC. Finally, the solution was filtered through 3 kDa Amicon Centrifugal Filters and stored at -20°C until HPLC-MSMS quantification analysis.

7. Mass spectrometry quantification

Filtered digestion products were thawed and serial dilutions were performed in MilliQ from x100 until x1600 dilution. 1 µl aliquots of the diluted samples were injected onto a 2.1 x 100 mm, 2.7 µm Agilent InfinityLab Poroshell 120 HILIC-Z column heated at 35°C. A binary gradient system consisting of A (10/90 v/v 10 mM Ammonium Formate in Formic acid-water solution, pH 3:water), and B (10/90 v/v 10 mM Ammonium Formate in Formic acid-water solution, pH 3:acetonitrile) was used. Sample separation was carried out at 0.8 ml/min over a 23 min total run time. The initial condition was 1/99 v/v A:B. The proportion of the solvent B was linearly decreased from 1/99 v/v A:B to 18/82 v/v A:B, from 0 min to 15 min, where the separation occurred. The rest of the run was meant to ensure proper washing of the system and avoid carryover. From 15 min to 16 min the composition went from 18/82 v/v A:B to 40/60 v/v A:B. Finally, the gradient was brought back to initial conditions linearly from minute 16 to minute 19, and let re-equilibrate until minute 23, where the pressure was back to the initial value. Detection was operated in positive ionization mode using the AJS Jet stream ESI source. AJS settings were as follows: Gas flow: 5 L/min; gas temperature: 300°C; nebulizer pressure, 45 psi; sheath gas temperature: 350°C; sheath gas flow: 11 L/min; capillary voltage:3500 V; Nozzle voltage: 500 V. The mass analyzer was used in the Multiple Reaction Monitoring (MRM). Transitions for the 20 AA were monitored as detailed in the table below.

AA	Prec. Ion (m/q)	Prod. Ion (m/q)	Frag (V)	CE (V)	Cell Acc. (V)	Set Ret. Time (min)	Window (min)	Obtained Ret. Time (min)
D	134	98.2	80	13	4	1.3	4	**1.38**
F	166.1	120	80	13	4	2	4	**1.82**
L	132.1	132.1	76	9	4	2.2	4	**1.97**
L	132.1	30.3	76	25	4	2.2	4	**1.97**
I	132.1	69.2	80	9	4	2.4	4	**2.15**
I	132.1	41.2	80	25	4	2.4	4	**2.15**
W	205.1	188	80	13	4	2.5	4	**2.20**
M	150.1	56.1	76	17	4	2.8	4	**2.45**
P	116.1	70.1	88	17	4	3.2	3	**2.82**
P	116.1	28.3	88	37	4	3.2	3	**2.82**
V	118.1	72.2	80	9	4	3.3	4	**2.84**
V	118.1	55.2	80	9	4	3.3	4	**2.84**
Y	182.1	119	80	17	4	3.5	4	**3.14**

C	122	59.1	80	37	4	4	4	**NA**
A	90.1	44.2	80	40	4	5	4	**4.59**
T	120.1	74.1	80	9	4	5.3	4	**4.87**
G	76	30.3	35	12	4	6	4	**5.50**
S	106.1	60.3	80	25	4	6.4	4	**5.99**
Q	147.1	84.1	80	17	4	6.2	4	**6.02**
N	133.1	74.2	80	17	4	6.7	4	**6.13**
E	148.1	102.1	80	17	4	8.4	4	**7.12**
H	156.1	110.1	90	13	4	11.9	5	**10.25**
R	175.1	70.1	80	24	4	11.7	4	**11.33**
K	147.1	130	80	17	4	12.9	4	**12.47**

The quantification of the samples was made by injecting standards just before and after the analysis of the samples, and then averaging the points to define the calibration curve. The standards were prepared as following: Individual AA stock solutions were manually prepared at 0.1 mg/mL in MilliQ and stored at -20°C. Before analysis, the stock solutions were mixed so to have a solution of the combined 20 AAs at 5 ug/mL each. The latter was further diluted to obtain the calibration standards (0.5 μg/mL, 0.25 μg/mL, 0.125 μg/mL, 62.5 ng/mL, 31.25 ng/mL, 15.62 ng/mL). Data were analyzed by using MassHunter Qualitative Analysis (Agilent Technologies. Inc.) to ensure correct identification of each amino acid, and using MassHunter Quantitative Analysis (Agilent Technologies. Inc.) to quantify the presence of each amino acid. A linear fit was computed for each amino acid using the integration results of the corresponding peak. Several dilutions of each sample were analyzed and those falling within the calibration range were taken for quantification. Standard deviations are calculated from technical duplicates.

8. Cell-free protein expression and purification

8.1 Cell-free expression of muGFP with commercial amino acids: 2 reactions from the RTS 500 ProteoMaster E Coli HY kit were reconstituted as detailed in the vendors protocol, split in volumes of 500 μL and introduced in four dialysis cups. Those were then introduced into the 5 mL Eppendorf tubes containing 4.5 mL of feeding mixture. Reactions were incubated in the thermomixer at 32°C for 24 hours and shaking at 600 rpm. After the incubation time, the reaction chambers were extracted with a pipet, ensuring resuspension by adding extra MilliQ water if needed, and merged into two 2mL tubes. Reactions were centrifuged at max speed for 15 minutes and the supernatant was recovered to perform purification.

8.2 Cell-free expression negative control: A reaction from the RTS 500 ProteoMaster E. Coli HY kit was reconstituted and assembled as detailed in the vendors protocol, but without introducing the amino acids neither in the reaction, nor in the feeding mix. Instead, we introduced the corresponding volume of Reconstitution Buffer. The reaction was split in volumes of 500 μL and introduced in dialysis cups. Those were then introduced into the 5 mL Eppendorf tubes containing 4.5 mL of feeding mixture. Reactions were incubated in the thermomixer at 32°C for 24 hours and shaking at 600 rpm. After the incubation time, the reaction chambers were extracted with a pipet, ensuring resuspension by adding extra MilliQ water if needed. Reactions were centrifuged at max speed for 15 minutes and the supernatant was recovered.

8.3 Cell-free expression of muGFP with recycled amino acids: 3 reactions from the RTS 500 ProteoMaster E Coli HY kit were reconstituted as detailed in the vendors protocol, but without introducing the amino acids neither in the reaction, nor in the feeding mix. Instead, we introduced the equivalent volume of recycled amino acids (from the digestion product). That is,

each of the three reactions was composed of: 0.525 mL of lysate, 0.225 mL of reaction mix, 0.3 mL of recycled amino acids, 15 μg of muGFP plasmid; and its Feeding mix was composed of: 8.1 mL of Feeding mix and 3 mL of recycled amino acids. Reactions were split in volumes of 500 μL and assembled into the dialysis devices and incubated as specified above. After the incubation time, the reaction chambers were extracted with a pipet, ensuring resuspension by adding extra MilliQ water if needed. Reactions were centrifuged at max speed for 15 minutes and the supernatant was recovered to perform purification.

8.4 Fluorescence measurement of the crude cell-free expression reaction: The supernatants were adjusted to equal volumes (if the reactions were resuspended by adding more water). Then, serial dilutions in MilliQ of 20 μL were introduced in a 384 well plate (Thermo Scientific # 242764 Black/Clear Bottom Plate), with duplicates, for plate reader fluorescence measurement of the expressed muGFP (Excitation: 480 nm, Emission: 509 nm). The measurement was performed after 10 seconds shaking, and the signal was measured from the bottom.

8.5 His-tag purification: Purification buffer A consisted of 700 mM of NaCl, 20 mM HEPES, and Purification buffer B consisted of 700 mM of NaCl, 20 mM HEPES, 500 mM Imidazole, both prepared using MilliQ water. 2 mL of Ni-NTA beads were equilibrated by washing two times with MilliQ water and two times with purification buffer A, by sedimenting and resuspending using centrifugation. The samples were further diluted to a final volume of 45 mL by addition of the corresponding amount of buffer A. The solution was then filtered with a 0.45 μm syringe filter (Chromafil Xtra PES-45/25) and mixed with the equilibrated Ni-NTA beads. 36 μL of imidazole solution (2.5 M) was added and the mixture was shacked gently for 1 hour. Gravity Glass Columns (Bio-Rad #7374251; 2.5 × 10 cm) were washed first with 70% ethanol, then with water, and then with buffer A prior to loading the incubated beads. During the elution process, all fractions were collected for a later inspection by SDS-PAGE gel electrophoresis. The sample was eluted by an imidazole gradient by mixing buffers A and B as following. Fraction 2: 50 mL of 20 mM imidazole, fraction 3: 50 mL of 50 mM imidazole, fraction 4: 10 mL of 100 mM imidazole, fraction 5, 6, 7, 8 and 9: 5 mL of 300 mM imidazole, fraction 10: 10 mL of 500 mM imidazole. Protein was found mostly in fraction 5, and in lesser amount in fraction 4, 6, and 7. Those fractions were mixed and dialyzed with a 3.5 kDa cutoff membrane (Sigma-Aldrich Pur-A-Lyzer Mega 3500, #PURG35010/15/25 the volume chosen accordingly in each experiment to fit the needed amount) against MilliQ water for 3 days, with five times exchanging of water, and later lyophilized.

8.6 Cell-free expressed muGFP hydrogel: The lyophilized protein was resuspended into 200 μL of MilliQ water to transfer it into a 2 mL Eppendorf tube and lyophilized again, for a more convenient handling. 0.5 mg of muGFP were recovered, as estimated by weighting the tube before and after. 4.1 μL of MilliQ water were added to solubilize the muGFP, followed by 0.45 μL of 100 mg/mL NHS and 0.45 μL of 100 mg/mL EDC (18%wt). The material was left to dry in the tube under the hood overnight. The morning after an insoluble material could be peeled off by the help of addition of MilliQ to plasticize it.

8.7 Cell-free expressed muGFP hydrogel with recycled amino acids: The tube containing the lyophilized recycled protein was whipped with ethanol and weighted in a microbalance. Then, the lyophilized protein was diluted and cross-linked as the control described in 3.6. This was an estimation because at that point we could not quantify the amount of protein we produced to adjust the fraction of EDC and NHS to add. The recycled protein cross-linked into a hydrogel when the NHS and EDC were introduced, and the material was left evaporate at room temperature overnight. The morning after, the recycled hydrogel was found dry, and it was recovered from the tube thanks to rehydrating it with MilliQ. To estimate the weight of the hydrogel, the tube was emptied, cleaned with ethanol and weighted to finally obtain the difference and estimate the amount of material produced.
